# Ocular manifestations of severe familial hypercholesterolemia^☆^^☆☆^

**DOI:** 10.1016/j.heliyon.2024.e30487

**Published:** 2024-04-30

**Authors:** Alaa Bou Ghannam, Rachid Istambouli, Mohamed S. Hamam, Jean M. Chalhoub, Akl C. Fahed, Rola N. Hamam

**Affiliations:** a Department of Ophthalmology, American University of Beirut Medical Center, Beirut, Lebanon; b Department of Internal Medicine, Massachusetts General Hospital, Harvard Medical School, Boston, MA, USA

**Keywords:** Corneal arcus, Fluorescein angiography, Hypercholesterolemia, Retinal vascular abnormalities

## Abstract

**Background:**

To study ocular manifestations of patients with severe familial hypercholesterolemia (FH).

**Methods:**

In this population-based case-control study, patients suffering from severe familial hypercholesterolemia from the Lebanese Familial Hypercholesterolemia Registry, along with age and gender-matched healthy controls were recruited. All participants underwent a comprehensive eye examination, and patients underwent fluorescein angiography as well. Logistic regression models were used to identify any association between patients with severe familial hypercholesterolemia and abnormal eye findings, while adjusting for hypertension and pack-year smoking. The main outcome measure of this study was the development of ocular vascular abnormalities.

**Results:**

28 patients and 28 controls were recruited. Patients with severe familial hypercholesterolemia had significantly greater odds of developing corneal arcus and xanthelasmas than the control group (p < 0.001). Retinal vascular abnormalities (plaques) were exclusively and more significantly present in patients with familial hypercholesterolemia (18 %). Similarly, retinal arteriosclerosis was exclusively and significantly more prevalent in the familial hypercholesterolemia group (p < 0.001, adjusted odds ratio 6.8). Stratification by LDL levels and genotypes did not show any significant change in the prevalence of any ocular finding.

**Conclusion:**

In addition to the well-established increase in incidence of corneal arcus and xanthelasmas, severe familial hypercholesterolemia patients have more prevalent retinal vascular abnormalities that include vascular plaques and arteriosclerosis.

## 1 Introduction

Familial Hypercholesterolemia (FH) is an inherited disease of cholesterol metabolism characterized by elevated Low-Density Lipoprotein (LDL) cholesterol in the serum. Heterozygous FH is common with a prevalence of 1:500 and is due to autosomal dominant mutations in the LDL receptor gene (*LDLR*). More rarely, a patient having both parents with heterozygous FH can inherit both mutated alleles, leading to the development of homozygous FH, which usually bears a more severe phenotype and has a prevalence of 1 in a million. The severe phenotype-defined as LDL levels above 190 mg/dl, regardless of the cause-can also be seen to a lesser extent in heterozygous carriers of *LDLR* mutations, and less commonly in patients with mutations in three other candidate genes: *ARH, Apo-B100*, and *PCSK9* [[1], [2], [3]]. The severe phenotype usually manifests with LDL levels that are more than three times the upper limit of normal. Patients exhibit external manifestations such as tendinous xanthomas, xanthelasmas, corneal arcus, and an accelerated onset of atherosclerosis and premature cardiovascular disease [1,4].

Ocular manifestations such as xanthelasmas and corneal arcus in patients with FH have been well documented in the literature [5]. However, reports of ocular vessel disease in severe FH patients are minimal. One recent study showed that patients with FH may have a larger foveal avascular zone [6].

There have been case reports showing a possible link between retinal vessel occlusion and hypercholesterolemia. In one such report, 3 out of 14 patients who had retinal vessel occlusion were found to have hypercholesterolemia [7,8]. In Lebanon, FH is at least a hundred times more prevalent than the worldwide average due to a genetic founder effect [1,9]. The Lebanese FH Registry has 25 families (124 subjects). This study aimed to define the prevalence of ocular abnormalities in patients with severe FH as compared to age and gender-matched controls.

## 2 Methods

The study was approved by the institutional review board (IRB) at the American University of Beirut (AUB) and all subjects signed informed consent. The study adhered to the tenants of Declaration of Helsinki. Between January 2012 and January 2013, 28 patients with severe FH were recruited from the Lebanese FH Registry. Each patient underwent a fasting lipid profile. Patients were then asked to fill out a questionnaire regarding demographics and pertinent medical history before undergoing a targeted physical examination including measurement of waist circumference, blood pressure, and body mass index calculation. Each patient then underwent a comprehensive slit lamp examination of the anterior segment and fundus by an ophthalmologist, including tear break-up time (TBUT) using fluorescein strips. Retinal arteriosclerosis was assessed and graded according to Sheie's classification [10]. Grade 1 arteriosclerosis was defined as minimal detectable widening of the arteriolar light reflex, while more significant widening with arteriovenous crossing sign represented grade 2. The presence of copper-wiring defined grade 3, while silver-wiring defined grade 4 arteriosclerosis. Those changes are thought to be due to arteriolar wall thickening and consequently retinal lumen arteriolar narrowing (RAN) [10]. All patients underwent fundus photography and fluorescein angiography. Fundus photographs and fluorescein angiograms were reviewed by two blinded ophthalmologists. Disagreements between the two observers were resolved by consensus.

Twenty-eight age and gender-matched healthy ds were recruited through IRB approved posters placed in AUB and public places surrounding the hospital area. Exclusion criteria for controls were any history of hypertension, diabetes mellitus, hypercholesterolemia, cardiovascular disease, and eye disease except for corrected refractive errors. A control subject's age was matched to within 2 years of a patient's age. Controls underwent a similar examination as FH patients excluding fluorescein angiography.

Genotype data obtained from a previous study [1] on the same patients was used to stratify patients according to mutation type. Patients having homozygous *LDLR* mutations (genotype 1) were contrasted to those having heterozygous *LDLR* mutations, recessive *LDLR* mutations, or mutations in other culprit genes (genotype 2).

Statistical analysis was performed using SPSS 22 (IBM, Chicago, IL). Logistic regression was performed to determine odds ratios, and adjusted odds ratios by adjusting for potential confounding variables (hypertension and pack-years of smoking). Stratification by LDL levels (LDL <400 mg/dL and LDL ≥400 mg/dL) was performed. Prevalence rates and associated 95 % confidence intervals were calculated for each abnormal eye finding detected in both patient and control groups. Differences in prevalence of ocular findings between the two groups were compared using the chi-square test. P-values less than 0.05 were considered significant.

## 3 Results

A total of 28 patients were recruited with the primary diagnosis of severe FH. Of these patients, 22 had undergone apheresis in the past with an average of 12 years of treatment. Patients had a mean LDL level of 438.5 mg/dL, with 13 patients (46 %) having LDL levels above 400 mg/dL. Demographic information of the patients and controls can be seen in Table 1. There was no significant difference between patients and controls with regards to social history (smoking, alcohol use) or markers of obesity (BMI and waist circumference). Stratifying patients by LDL levels (greater or less than 400 mg/dL) showed no significant differences in social history, coronary artery disease or hypertension between the two groups.

**Table 1:** Baseline characteristics for familial hypercholesterolemia patients with LDL level stratification and controls.

	Controls (n = 28)		Patients General (n = 28)		p value	Patients LDL <400 mg/dL (n = 15)		Patients LDL ≥400 mg/dL (n = 13)		p value
Female	16	(57)	16	(57)		9	(60)	7	(54)	0.74
Mean age, yrs (SD)	29	(13)	29	(13)		30	(16)	27	(9)	0.17
Current smoker	7	(25)	12	(43)	0.16	6	(40)	6	(46)	0.74
Pack-year (SD)	0.04	(0.2)	4.2	(12)	0.067	7	(16)	1	(3)	0.23
Alcohol use	9	(32)	12	(43)	0.41	8	(53)	4	(31)	0.23
BMI (SD)	25	(4)	24	(4)	0.17	23	(4)	24	(3)	0.26
Waist circumference (SD)	90	(26)	86	(13)	0.38	85	(15)	87	(11)	0.66
LDL level, mg/dL (SD)	101	(27)	438	(141)	<0.0001	320	(83)	548	(82)	<0.0001
Past Medical History										
Hypertension	0	(0)	4	(14)	0.04	2	(13)	2	(15)	0.88
Diabetes mellitus	0	(0)	1	(4)	0.31	1	(7)	0	(0)	0.34
Coronary artery disease	0	(0)	9	(32)	0.001	6	(40)	3	(23)	0.34
Mean systolic BP, mmHg (SD)	118	(12)	124	(13)	0.12	119	(11)	124	(14)	0.69
Mean diastolic BP, mmHg (SD)	77	(9)	67	(13)	0.004	63	(10)	68	(12)	0.39
Current or past apheresis			22	(79)		10	(67)	12	(92)	0.10
Years on apheresis (SD)			12	(7)		11	(8)	13	(6)	0.40
Apheresis rate, days (SD)			18	(11)		17	(7)	18	(13)	0.89

SD = standard deviation.

BMI = body mass index.

LDL = low-density lipoprotein.

BP = blood pressure.

Data are in n (%) unless otherwise indicated.

**Fig. 1:**
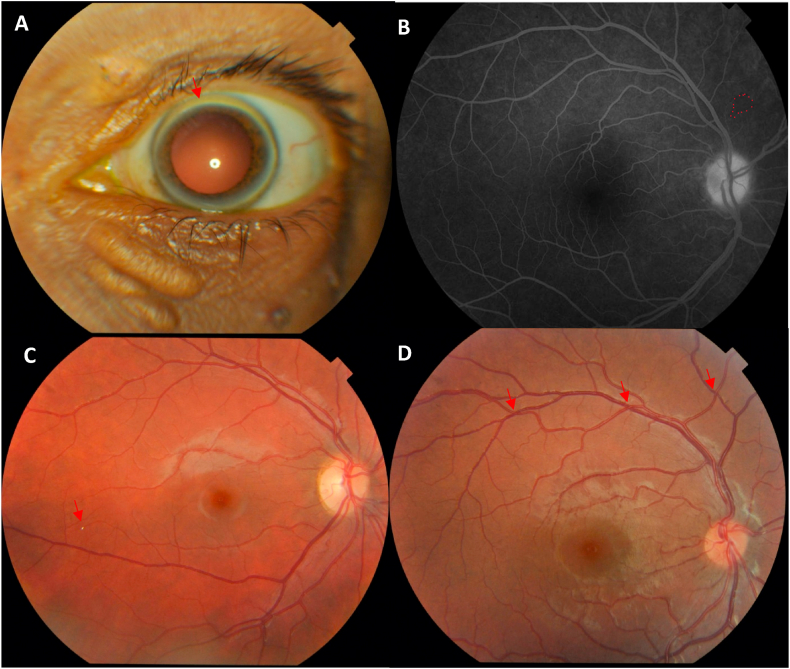
**A.** Corneal Arcus in a patient with severe familial hypercholesterolemia **B.** A case of a branch retinal artery occlusion (BRAO) observed in a small nasal branch of the retinal artery in fundus fluorescein angiography (FA) **C.** A case of Grade I retinal arteriosclerosis by Sheie classification with a visible Hollenhorst plaque in fundus photography (FP) **D.** A case of Grade II retinal arteriosclerosis by Sheie classification in fundus photography (FP).

Ocular findings in patients and controls are presented in Table 2, Table 3. Decreased TBUT was found to be similar in patients and controls. Patients with severe FH had 83.1 (95 % CI, 8.9–776.5; p < 0.001) times greater odds of developing corneal arcus (Fig. 1A) than the control group. Patients were also found to have significantly higher prevalence of xanthelasmas than the control group (32 % compared to 0 % in controls; p = 0.001). Two retinal arterial plaques and two retinal venous plaques were observed in four patients with FH (Fig. 1B). Retinal arteriosclerosis was significantly more prevalent in the FH group (p < 0.001, adjusted odds ratio 6.8). We observed four controls and 12 FH patients with grade 1 arteriosclerosis (Fig. 1C). Additionally, six FH patients had grade 2 arteriosclerosis (Fig. 1D), while we found none in the control group.

**Table 2:** Ocular findings for familial hypercholesterolemia patients and controls with multivariate logistic regression.

	Control (n = 28)		Patients General (n = 28)		p-value	Odds Ratio (95 % CI)		Adjusted Odds Ratio (95 % CI)	
Corneal arcus	1	(4)	18	(64)	<0.001*	48.6	(5.7–413.2)	83.1	(8.9–776.5)
Lipid deposits or xanthelasmas	0	(0)	9	(32)	0.001*				
Excision of xanthomas	0	(0)	8	(29)	0.004*				
TBUT <10 s	11	(42)	12	43	1.0	1.0	(0.4–3.0)	1.1	(0.4–3.6)
Occluded retinal arteries	0	(0)	1	(4)	0.31				
Retinal arterial plaque	0	(0)	2	(7)	0.15				
Retinal venous plaque	0	(0)	2	(7)	0.15				
Retinal arteriosclerosis	4	(14)	18	(64)	<0.001*	10.8	(2.9–40.1)	6.8	(1.6–27.8)
Grade 1	4	(14)	12	(43)					
Grade 2	0	(0)	6	(21)					

CI = confidence interval.

LDL = low-density lipoprotein.

TBUT = tear break-up time.

Data are in n (%) unless otherwise indicated.

**Table 3:** Ocular findings for familial hypercholesterolemia patients stratified according to LDL levels with multivariate logistic regression.

	Patients LDL <400 mg/dL (n = 15)		Patients LDL ≥400 mg/dL (n = 13)		p-value	Odds Ratio: LDL <400 mg/dL vs. LDL ≥400 mg/dL (95 % CI)		Adjusted Odds Ratio: LDL <400 mg/dL vs. LDL ≥400 mg/dL (95 % CI)	
Corneal arcus	7	(47)	11	(85)	0.055	6.3	(1.0–38.6)	10.4	(0.4–61.2)
Lipid deposits or xanthelasmas	3	(20)	6	(46)	0.23	1.2	(0.2–7.3)	1.1	(0.1–9.1)
Excision of xanthomas	2	(13)	6	(46)	0.10	5.6	(0.9–35.3)	11.7	(1.0–140.8)
TBUT ≤10 s	6	(40)	6	(46)	1.0	1.3	(0.3–5.8)	0.5	(0.1–2.9)
Occluded arteries	1	(7)	0	(0)	0.34				
Retinal arterial plaque	0	(0)	2	(15)	0.12				
Retinal venous plaque	2	(13)	0	(0)	0.17				
Retinal arteriosclerosis	10	(67)	8	(62)	1.0	0.8	(0.2–3.8)	0.93	(0.18–4.8)
Grade 1	7		5		0.9				
Grade 2	3		3						

CI = confidence interval.

LDL = low-density lipoprotein.

TBUT = tear break-up time.

Data are in n (%) unless otherwise indicated.

Stratification by LDL levels did not show any significant change in the prevalence of any ocular finding. Twelve patients were known to have homozygous *LDLR* mutations, while 9 patients had either the recessive or a heterozygous *LDLR* genotype (Table 4). Similar mean LDL levels were found in the different mutations, and no significant difference between the ocular findings was observed.

**Table 4:** Ocular findings by Patient Genotype.

	Genotype 1 (n = 12)			Genotype 2 (n = 9)			p-value	Odds Ratio: Dominant vs. Recessive genotype (95 % CI)	
LDL level, mg/dL (SD)	455	(142)		444	(122)		0.85		
Arcus	9	(75)		3	(33)		0.06	6.00	(0.89–40.31)
TBUT <10 s	5	(42)		5	(56)		0.53	1.75	(0.31–10.02)
Retinal arteriosclerosis	5	(42)		8	(89)		0.067	11.2	(1.04–120)
Grade 1	3	(25)		4	(45)				
Grade 2	2	(17)		4	(45)				

Genotype 1 = homozygous *LDLR* mutation.

Genotype 2 = heterozygous *LDLR* mutation, recessive *LDLR* mutation, other genes involved.

CI = confidence interval.

LDL = low-density lipoprotein.

TBUT = tear break-up time.

Data are in n (%) unless otherwise indicated.

## 4 Discussion

Results from this study show that ocular changes are more prevalent among patients with severe FH. Many of these findings have been previously reported in the literature, namely corneal arcus and xanthelasma [5]. Here we report a higher prevalence of retinal vascular abnormalities in patients with severe FH.

Animal studies have shown that rats with hypercholesterolemia have microscopic changes in the retinal vasculature [11]. In FH patients, it has been demonstrated that LDL apharesis causes change in diameter and improvement in flicker-induced vasodilation of retinal vessels [12,13]. Recent studies using OCTA have shown that patient with FH may have a larger foveal avascular zone [6]. Here we show that vascular changes in severe FH can also be detected on regular fundus examination, using characteristics of the arteriolar light reflex. By applying the Sheie classification of hypertensive arteriosclerosis, we found that patients with severe FH have more significant retinal arteriosclerosis than age and gender-matched controls, independently of hypertension and pack-years of smoking (adjusted odds ratio = 6.8). Furthermore, 21 % of the FH cohort had grade 2 arteriosclerosis, signifying more marked arteriolar wall stiffening and arteriovenous compression. Several studies investigated the association of retinal microvascular changes and coronary artery diseases. RAN proved to be a predictor of microvascular dysfunction and is associated with chronic hypertension as well as arterial stiffness [[14], [15], [16]]. Yet, regardless of the hypertensive state of an individual, narrowing of the retinal vessel lumen has been associated with a higher incidence of cardiovascular events [17,18]. A longitudinal study by Kawashima-Kumagai et al. followed up more than 6000 individuals and documented retinal arteriolar changes over five years and concluded that patients who develop RAN regardless of their blood pressure changes are at a higher risk of developing large vessel systemic arteriosclerosis. The latter is a known risk factor for developing coronary artery diseases [19]. Given the above, it might be advisable to do regular retinal exams in patients with FH looking for early signs of arteriosclerosis using the Sheie classification which could be a predictor of their systemic arteriosclerosis state and risk of cardiovascular events.

Four patients were found to have plaques involving the retinal vessels, two of which involved arteries while the other two involved veins. In one eye, we observed occlusion of a small branch of the retinal artery. Retinal vascular plaques are associated with retinal vessel occlusions and stroke, although their presence alone does not predict such events in the future [20]. A possible explanation could be embolization from the carotid artery, as it is well established that FH patients are susceptible to carotid artery atherosclerosis [1]. In our patient cohort, six patients had carotid plaques as per carotid ultrasound findings, and four patients had a carotid stenosis >60 %. However, we found no correlation between percentage of carotid stenosis, presence of carotid plaques, or carotid intima-media thickness with retinal vascular pathology in our cohort. Of the four patients who had retinal vascular plaques, only one of them had carotid plaques as per carotid ultrasound findings.

Additionally, as demonstrated by other studies, we observed corneal arcus in a significant proportion of FH patients [5,21,22]. We did not, however, find any difference in the tear break-up time between FH patients and controls. There is conflicting data concerning the association between dyslipidemia and both Meibomian gland dysfunction and dry eye syndrome [[23], [24], [25], [26]]. In our cohort of FH patients, the prevalence of ocular surface dryness seemed to be equal to that in controls.

Whether the observed ocular changes are reversible cannot be concluded from our cross-sectional study. All our FH patients were already on treatment at the time of the ophthalmologic examination. Animal models have shown that cholesterol is deposited in the uveal tract of the eye and that vascular changes can be observed in the choroid [27,28]. Normalization of serum cholesterol was not enough to reverse cholesterol-induced vascular damage to the choroid, which may be due to the fact that despite therapy, patients have an increased expression of several inflammatory factors of TNF-related genes [29]. This potentially explains the similar prevalence of ocular changes in FH patients with LDL levels greater or less than 400 mg/dL. Finally, ocular findings did not differ by mutation type (dominant vs. recessive), as would be expected if the ocular findings observed are a result of a shared common pathway of high serum LDL.

A limitation of our study is its cross-sectional nature. We report changes in the retinal vasculature of FH patients, including retinal arteriosclerosis and vascular plaques. These vascular changes are associated with sight-threatening vascular events. A longitudinal study of such a cohort of FH patients would elucidate if these lesions predict vision loss in these patients over their lifetime. Moreover, the findings of this study are relevant to patients with FH that have very high LDL-C levels, and are not generalizable to patients with standard hyperlipidemia.

In conclusion, we found a higher prevalence of corneal arcus and xanthelasma in FH patients. Additionally, patients with FH had more prevalent and more severe retinal arteriosclerosis, as well as retinal vascular plaques. Our findings may signify the need for routine eye examination in FH patients, looking for early signs of arteriosclerosis using the Sheie classification which could be a predictor for systemic arteriosclerosis state and risk of cardiovascular events. This will need further investigation in future prospective studies.

## Data availability statement

Data associated with the study has not been deposited into a publicly available repository, as patient data used in this manuscript is confidential.

## Ethics statement

The study was approved by the institutional review board (IRB) at the American University of Beirut (AUB) (IRB Protocol Number OPH.RH.02) and all subjects signed informed consent. The study adhered to the tenants of Declaration of Helsinki.

## CRediT authorship contribution statement

**Alaa Bou Ghannam:** Writing – review & editing, Writing – original draft, Formal analysis, Data curation. **Rachid Istambouli:** Writing – review & editing, Writing – original draft, Formal analysis, Conceptualization. **Mohamed S. Hamam:** Writing – original draft, Formal analysis, Data curation, Conceptualization. **Jean M. Chalhoub:** Writing – original draft, Methodology, Formal analysis, Data curation, Conceptualization. **Akl C. Fahed:** Writing – review & editing, Writing – original draft, Methodology, Formal analysis, Data curation. **Rola N. Hamam:** Writing – review & editing, Writing – original draft, Supervision, Methodology, Funding acquisition, Formal analysis, Data curation, Conceptualization.

## Declaration of competing interest

No conflicting relationship exists for any author.

## References

[1] Fahed A.C., Safa R.M., Haddad F.F. (2011). Homozygous familial hypercholesterolemia in Lebanon: a genotype/phenotype correlation. Mol. Genet. Metabol..

[2] Hopkins P.N., Toth P.P., Ballantyne C.M., Rader D.J. (2011). Familial hypercholesterolemias: prevalence, genetics, diagnosis and screening recommendations from the national lipid association expert panel on familial hypercholesterolemia. J Clin Lipidol.

[3] Al-Hinai A.T., Al-Abri A., Al-Dhuhli H. (2013). First case report of familial hypercholesterolemia in an Omani family due to novel mutation in the low-density lipoprotein receptor gene. Angiology.

[4] Khachadurian A., Uthman S. (1973). Experiences with the homozygous cases of familial hypercholesterolemia: a report of 52 patients. Nutr. Metab..

[5] Blodi F.C., Yarbrough J.C. (1962). Ocular manifestations of familial hypercholesterolemia. Trans. Am. Ophthalmol. Soc..

[6] Stefanutti C., Mesce D., Pacella F. (2019). Optical coherence tomography of retinal and choroidal layers in patients with familial hypercholesterolaemia treated with lipoprotein apheresis. Atherosclerosis Suppl..

[7] Dodson P., Kubicki A., Taylor K., Kritzinger E. (1985). Medical conditions underlying recurrence of retinal vein occlusion. Br. J. Ophthalmol..

[8] Dodson P., Galton D., Hamilton A., Blach R. (1982). Retinal vein occlusion and the prevalence of lipoprotein abnormalities. Br. J. Ophthalmol..

[9] Lehrman M.A., Schneider W., Brown M. (1987). The Lebanese allele at the low density lipoprotein receptor locus. Nonsense mutation produces truncated receptor that is retained in endoplasmic reticulum. J. Biol. Chem..

[10] Scheie H.G. (1953). Evaluation of ophthalmoscopic changes of hypertension and arteriolar sclerosis. AMA Arch Ophthalmol.

[11] Yamakawa K., Ahmed Bhutto I., Lu Z., Watanabe Y., Amemiya T. (2001). Retinal vascular changes in rats with inherited hypercholesterolemia–corrosion cast demonstration. Curr. Eye Res..

[12] Terai N., Julius U., Haustein M., Spoerl E., Pillunat L.E. (2011). The effect of low-density lipoprotein apheresis on ocular microcirculation in patients with hypercholesterolaemia: a pilot study. Br. J. Ophthalmol..

[13] Reimann M., Prieur S., Lippold B. (2009). Retinal vessel analysis in hypercholesterolemic patients before and after LDL apheresis. Atherosclerosis Suppl..

[14] Fuchs S.C., Pakter H.M., Maestri M.K. (2015). Are retinal vessels calibers influenced by blood pressure measured at the time of retinography acquisition?. PLoS One.

[15] Leung H., Wang J.J., Rochtchina E., Wong T.Y., Klein R., Mitchell P. (2004). Impact of current and past blood pressure on retinal arteriolar diameter in an older population. J. Hypertens..

[16] Triantafyllou A., Anyfanti P., Gavriilaki E. (2014). Association between retinal vessel caliber and arterial stiffness in a population comprised of normotensive to early-stage hypertensive individuals. Am. J. Hypertens..

[17] Mutlu U., Ikram M.K., Wolters F.J., Hofman A., Klaver C.C., Ikram M.A. (2016). Retinal microvasculature is associated with long-term survival in the general adult Dutch population. Hypertension.

[18] Wong T.Y., Klein R., Nieto F.J. (2003). Retinal microvascular abnormalities and 10-year cardiovascular mortality: a population-based case-control study. Ophthalmology.

[19] Kawashima-Kumagai K., Tabara Y., Yamashiro K. (2018). Association of retinal vessel calibers and longitudinal changes in arterial stiffness: the Nagahama study. J. Hypertens..

[20] Kaufman E.J.M.N., Patel B.C. (2020). Hollenhorst Plaque.

[21] Zech L.A., Hoeg J.M. (2008). Disease. Correlating corneal arcus with atherosclerosis in familial hypercholesterolemia. Lipids Health Dis..

[22] Lock J.H., Ross C.A., Flaherty M. (2018). Corneal arcus as the presenting sign of familial hypercholesterolemia in a young child. J AAPOS.

[23] Guliani B.P., Bhalla A., Naik M.P. (2018). Association of the severity of meibomian gland dysfunction with dyslipidemia in Indian population. Indian J. Ophthalmol..

[24] Módulo C.M., Filho E.B.M., Malki L.T. (2012). The role of dyslipidemia on ocular surface, lacrimal and meibomian gland structure and function. Curr. Eye Res..

[25] Pinna A., Blasetti F., Zinellu A., Carru C., Solinas G. (2013). Meibomian gland dysfunction and hypercholesterolemia. Ophthalmology.

[26] Kuriakose R.K., Braich P.S. (2018). Dyslipidemia and its association with meibomian gland dysfunction: a systematic review. Intl Ophthalmol.

[27] Salazar J.J., Ramírez A.I., de Hoz R. (2007). Alterations in the choroid in hypercholesterolemic rabbits: reversibility after normalization of cholesterol levels. Exp. Eye Res..

[28] Kouchi M., Ueda Y., Horie H., Tanaka K. (2006). Ocular lesions in Watanabe heritable hyperlipidemic rabbits. Vet. Ophthalmol..

[29] Holven K.B., Narverud I., Lindvig H.W. (2014). Subjects with familial hypercholesterolemia are characterized by an inflammatory phenotype despite long-term intensive cholesterol lowering treatment. Atherosclerosis.

